# *Epilepsia Open*—August 2024 Announcements

**DOI:** 10.1002/epi4.13017

**Published:** 2024-08-02

## ILAE CONGRESSES

15th European Epilepsy Congress

September 7–11, 2024

Rome, Italy

14th International Summer School for Neuropathology and Epilepsy Surgery

September 19–22, 2024

Erlangen, Germany

Advanced Pediatric Epilepsy Surgery Course 2024

November 7–10, 2024

Cochin, India

7th East Mediterranean Epilepsy Congress

November 14–16, 2024

Manama, Bahrain

## 2025

15th Asian & Oceanian Epilepsy Congress

February 20–23, 2025

New Delhi, India

36th International Epilepsy Congress

August 30–September 3, 2025

Lisbon, Portugal

## ILAE WEBINARS

ILAE e‐Forum: Co‐morbidities and mental health in adults with epilepsy

August 1, 2024

Management of Neonatal Seizures

6 August 2024

Démarche diagnostique devant une épilepsie

13 August 2024

ILAE e‐Forum: Reducing epilepsy related mortality

September 23, 2024

Psychosocial Factors and Approaches to Care

25 September 2024

ILAE e‐Forum: Optimal timing for epilepsy surgery

November 25, 2024

## OTHER CONGRESSES

Primer Congreso Paraguayo de Epilepsia

August 1–2, 2024

Asunción, Paraguay

XLVI Reunión Anual del Capítulo Mexicano de la Liga Internacional Contra la Epilepsia

August 6–10, 2024

Monterrey, Nuevo León, Mexico

Curso teórico práctico de EEG y epilepsia en pediatría

August 9–10, 2024

Santiago, Chile

17th Baltic Sea Summer School on Epilepsy

August 15–September 2, 2024

Online

Localisation en EEG

September 6, 2024

Rabat, Maroc

33rd International Congress of Clinical Neurophysiology

10–14 September 2024

Jakarta, Indonesia

Cleveland Clinic International Epilepsy Summit

11–15 September 2024

Cleveland, Ohio, USA

10th Eilat International Educational Course: Pharmacological Treatment of Epilepsy

September 15–20, 2024

Limassol, Cyprus

16th International Epilepsy Course and Colloquium

September 17–20, 2024

Frankfurt am Main, Germany

World Federation of Neurology Digital Neurology Updates

26–27 September 2024

Online

6th Bologna EPIPED‐EEG Course: EEG interpretation in pediatric epilepsies

September 29–October 3, 2024

Bologna, Italy

26èmes Journées Françaises de l'Epilepsie

October 8–11, 2024

Marseille, France

Epilepsy Training Course

10–11 October 2024

Medan, Indonesia

Cours épilepsie et EEG pour les médecins de première ligne

11–12 October 2024

Sfax, Tunisie

Cairo—Epilepsy Workshop

October 16, 2024

Cairo, Egypt

Epilepsy Training Course

October 18–20, 2024

Douala, Cameroon

SEEG Course 2024

October 31–November 2, 2024

Thailand

2nd Annual Canadian Paediatric SEEG Conference

1–3 November 2024

London, Ontario, Canada

Epilepsy Society of Australia Annual Scientific Meeting 2024

November 6–8, 2024

Hobart, Tasmania, Australia

2nd Advanced Course on the Pharmacological Treatment of Drug Resistant Epilepsies (DRE): From Rare to Common Epilepsies

8–10 November 2024

Palma de Mallorca, Spain

Encephalitis 2024

December 2–3, 2024

London, UK & Online

AES 2024 Annual Meeting

December 6–10, 2024

Los Angeles, California, USA

7th East Mediterranean Epilepsy Congress

12–15 December 2024

Baghdad, Iraq

## 2025

19th World Congress on Controversies in Neurology

20–22 March 2025

Prague, Czech Republic

International Congress on Structural Epilepsy & Symptomatic Seizures 2025

April 2–4, 2025

Gothenburg, Sweden

11th Congress of the European Academy of Neurology

June 21–25, 2025

Sevilla, Spain

19th European Congress for Clinical Neurophysiology

9–12 September 2025

London, UK

## 2026

18th Eilat Conference on New Antiepileptic Drugs and Devices

May 3–6, 2026

Madrid, Spain

